# ACOT12, a novel factor in the pathogenesis of kidney fibrosis, modulates ACBD5

**DOI:** 10.1038/s12276-025-01406-3

**Published:** 2025-02-13

**Authors:** Ee Hyun Kim, Mi Kyung Kim, MiSun Choe, Ji Hyun Ryu, Eun Seon Pak, Hunjoo Ha, Eun-Jung Jin

**Affiliations:** 1 Graduate School of Pharmaceutical Sciences, College of Pharmacy, Ewha Womans University, Seoul, South Korea; 2 Integrated Omics Institute, Wonkwang University, Iksan, South Korea; 3 Department of Internal Medicine, School of Medicine, Keimyung University, Daegu, South Korea; 4 Department of Pathology, School of Medicine, Keimyung University, Daegu, South Korea; 5 Department of Biomedical Materials Science, Graduate School of JABA, Wonkwang University, Iksan, South Korea

**Keywords:** Chronic kidney disease, Diagnostic markers, Pathogenesis, Biomarkers

## Abstract

Lipid metabolism, particularly fatty acid oxidation dysfunction, is a major driver of renal fibrosis. However, the detailed regulatory mechanisms underlying this process remain unclear. Here we demonstrated that acyl-CoA thioesterase 12 (Acot12), an enzyme involved in the hydrolysis of acyl-CoA thioesters into free fatty acids and CoA, is a key regulator of lipid metabolism in fibrotic kidneys. A significantly decreased level of ACOT12 was observed in kidney samples from human patients with chronic kidney disease as well as in samples from mice with kidney injuries. Acot12 deficiency induces lipid accumulation and fibrosis in mice subjected to unilateral ureteral obstruction (UUO). Fenofibrate administration does not reduce renal fibrosis in *Acot12*^*−/−*^ mice with UUO. Moreover, the restoration of peroxisome proliferator-activated receptor α (PPARα) in *Acot12*^*−/−*^*Pparα*^*−/−*^ kidneys with UUO exacerbated lipid accumulation and renal fibrosis, whereas the restoration of Acot12 in *Acot12*^*−/−*^ *Pparα*^*−/−*^ kidneys with UUO significantly reduced lipid accumulation and renal fibrosis, suggesting that, mechanistically, *Acot12* deficiency exacerbates renal fibrosis independently of PPAR*α*. In *Acot12*^*−/−*^ kidneys with UUO, a reduction in the selective autophagic degradation of peroxisomes and pexophagy with a decreased level of ACBD5 was observed. In conclusion, our study demonstrates the functional role and mechanistic details of Acot12 in the progression of renal fibrosis, provides a preclinical rationale for regulating Acot12 expression and presents a novel means of preventing renal fibrosis.

## Key role of Acot12 in kidney fibrosis revealed

Chronic kidney disease (CKD) is a serious condition that affects kidney function over time. It can lead to other health problems, but current treatments are limited. Here the authors wanted to explore new ways to treat CKD by focusing on kidney fibrosis, a major factor in CKD progression. This study demonstrates that decreased level of ACOT12, a critical enzyme in lipid metabolism, correlates with increased fibrosis and lipid accumulation in the kidney tissues. Mice lacking ACOT12 had more kidney fibrosis and lipid accumulation, suggesting that ACOT12 plays a protective role, while restoring ACOT12 in these mice reduced fibrosis and lipid buildup. These findings suggest that targeting ACOT12 could be a novel therapeutic approach for preventing kidney fibrosis by regulating lipid metabolism in CKD.

This summary was initially drafted using artificial intelligence, then revised and fact-checked by the author.

## Introduction

Chronic kidney disease (CKD) is a progressive disorder characterized by changes in kidney structure, such as cysts, tumors and atrophy and/or loss of kidney function, over a period of months to years. Kidney dysfunction can change the output or quality of urine and is most often recognized by increased serum levels of creatinine, cystatin C or blood urea nitrogen (BUN). Patients with CKD have an increased risk of developing other complications, such as cardiovascular diseases, hypertension and bone disorders. Although the impact of CKD on morbidity and mortality is increasing worldwide^1^, there is no effective treatment for CKD other than dialysis or kidney transplantation.

Kidney fibrosis is the final common pathway in CKD and is characterized by glomerulosclerosis, tubulointerstitial fibrosis, inflammatory infiltration and kidney parenchyma loss. Excessive accumulation of the extracellular matrix, which is primarily composed of collagen, results in loss of kidney function^2,3^, and impeding or reversing this process is one of the current therapeutic approaches for controlling CKD^4^. Recently, peroxisome proliferator-activated receptors (PPARs) have been implicated in the regulation of kidney inflammation and fibrosis and are considered potential therapeutic targets^5^. PPARα regulates age-associated kidney fibrosis^6^. PPARα deficiency leads to the accumulation of lipids in kidney tubules and exposure to PPARα agonists reduces the production of transforming growth factor-beta (TGFβ), IL-1β, IL-6 and TNF, leading to a reduction in tubulointerstitial fibrosis and inflammation^7,8^. Moreover, PPARγ, which is known to be expressed in glomerular mesangial cells, podocytes and proximal epithelial cells, has a protective role against acute kidney injury by inhibiting inflammation, and pioglitazone, a PPARγ agonist, prevents mesangial expansion, glomerulosclerosis, tubulointerstitial inflammation and fibrosis, as well as tubular dilation and atrophy during the pathogenesis of diabetic nephropathy^9,10^.

Increasing evidence supports the hypothesis that lipid abnormalities contribute to the progression of kidney fibrosis^11^. Fatty acid oxidation (FAO) is closely associated with the development and progression of fibrosis because the kidneys mostly rely on fatty acid (FA) β-oxidation as their energy source. Thus, FAO dysfunction can exacerbate kidney fibrosis. Carnitine palmitoyltransferase 1A (CPT1A), a rate-limiting enzyme in the FAO pathway, is expressed in kidney tubules and is significantly reduced in kidney fibrosis^12^. FA transporters in the kidney, such as clusters of differentiation-36 (CD36), FA-binding proteins and FA-transport proteins, also play important roles in kidney disease. CD36, a FA translocase, is involved in Wnt/β-catenin activation and contributes to lipid accumulation and kidney fibrosis^13,14^. CD36 is associated with the collagen I-discoidin domain receptor 1 pathway and causes kidney failure in a collagen 4-mutated lipotoxic injury model^15^. Inhibition of FABP4 attenuates endoplasmic reticulum stress, mitochondrial dysfunction and inflammation in kidney fibrosis^16,17^. FATP2, encoded by Slc27a2, promotes kidney fibrosis by inhibiting FAO^18^. These data suggest that lipid metabolism accompanying CKD in kidney fibrosis could be a potential therapeutic target. Given the critical role of lipid metabolism in kidney fibrosis, we focused on acyl-CoA thioesterase 12 (ACOT12) in this study. ACOT12 is an enzyme that hydrolyzes acyl-CoA to free FAs and CoA, which is crucial in regulating the intracellular levels of acyl-CoA and free FAs. It is predominantly expressed in the kidney tubules, suggesting its potential involvement in renal lipid metabolism. However, the role of ACOT12 in kidney fibrosis has not been previously explored. We hypothesized that ACOT12 might be a key regulator linking lipid metabolism to kidney fibrosis. Our results demonstrate that *Acot12* deficiency induces lipid accumulation and exacerbates kidney fibrosis independently of PPARα, and that restoring *Acot12* effectively ameliorates kidney fibrosis.

## Materials and methods

### Ethical approval

All animal studies were approved by the Institutional Animal Care and Use Committee of Wonkwang University and followed institutional guidelines (WKU20-60, WKU20-114, WKU21-36, WKU21-89 and WKU22-40). All the mice were housed at 23 ± 1 °C for 12 h. The light/dark cycles and relative humidity were 50 ± 5%, with food and water available ad libitum. Human kidney tissues were obtained from 12 patients with chronic obstructive nephropathy and 17 patients with clear cell renal cell carcinoma. The study protocol was approved by the clinical research ethics committee of Keimyung University Dongsan Hospital (institutional review board no. 2022-08-022), and all experiments were performed following approved guidelines.

### Animal experiments

Global *Acot12*^*−/−*^ mice were generated via the germline transmission of an RGEN-induced mutant allele^19^. *Acot12*^*−/−*^*PPARα*^*−/−*^ double knockout (KO) mice were generated by crossbreeding. Male 6–7-week-old C57BL/6N mice underwent unilateral ureteral obstruction (UUO) surgery. UUO surgery was performed under anesthesia, as previously described^20^, and the kidneys were collected on the seventh day. Fenofibrate (125 mg/kg) was orally administered once daily from three days before UUO surgery to the day before killing. For the adenine diet model, the mice were fed a 0.25% adenine diet for 2 weeks.

### Lentiviral packaging

Lenti-X 293T cells (Clontech) were transfected with pLenti-GIII-CMV-*Acot12* or *pLenti-GIII-CMV-PPARα*pLenti-GIII-CMV-*PPAR*α via a third-generation packaging system mix (Applied Biosystems) according to the manufacturer’s protocol. The lentiviral particles were concentrated via a Lenti-X concentrator (Takara) and stored at −80 °C. The lentiviruses were injected into four sites in the kidney parenchyma after UUO surgery.

### Synthesis of CHI–DHCA

Catechol-modified chitosan (CHI–DHCA) was synthesized via standard carbodiimide chemistry. Briefly, chitosan (0.5 g) (molecular weight of 100 kDa, 70% deacetylated, Heppe Medical Chitosan, GmbH) was dissolved in distilled water (44.5 ml) containing 5 ml of 1 N HCl. After the complete dissolution of the chitosan, the pH was adjusted to 5 with 1 N NaOH. Then, 3,4-dihydroxyhydrocinnamic acid (DHCA, 0.59 g) (Sigma‒Aldrich) and 1-ethyl-3-(3-dimethylaminopropyl)carbodiimide (0.62 g) (TCI-SU) in ethanol (12.5 ml) were added slowly to the chitosan solution and allowed to react for 12 h. The pH was adjusted to 5.0 using 1 N HCl to prevent the undesirable oxidation of the catechol groups during the reactions. The product was purified via a dialysis membrane (molecular weight cutoff of 12–14 kDa; SpectraPor) against a pH 2 NaCl (10 mM) solution for 2 days, distilled water for 4 h and subsequently lyophilized. The degree of catechol substitution was confirmed via ^1^H nuclear magnetic resonance (Bruker Avance, 500 MHz) and ultraviolet‒visible (UV–Vis) spectroscopy (UV-1900i, Shimadzu). The degree of DHCA substitution of CHI–DHCA was calculated from the absorbance at 280 nm with standard curves of DHCA monomer concentrations.

### Generation of Acot12-conjugated CHI–DHCA patches

Chitosan-catechol (40 mg/ml) was mixed with 50 μg of pcDNA-RFP-*Acot12*, lyophilized for 1 day and used for the delivery of *Acot12* into the kidney.

### PTEC culture

Kidney cortical fragments isolated from 3-week-old male C57BL/6 mice were digested with collagenase type I (Thermo Fisher Scientific) and filtered through a 100 μm strainer^12^. After red blood cells were lysed with RBC lysis buffer (Roche), the primary tubule epithelial cells (PTECs) were centrifuged, washed twice with RPMI 1640 and grown in RPMI 1640 (Gibco) supplemented with 20 ng/ml recombinant human epidermal growth factor (Sigma) and 10% fetal bovine serum.

### GFP-LC3/RFP-SKL transfection

PTECs were transfected with plasmids encoding *GFP-LC3* and *RFP-SKL* via Lipofectamine 3000 (Invitrogen) in the presence of 5 ng/ml TGFβ and 10 nM bafilomycin A for 24 h, and fluorescence images were acquired via EVOS FL Auto software v.1.7.

### Histology and immunohistochemistry

The kidneys were fixed in 4% paraformaldehyde (pH 7.4), dehydrated and embedded in paraffin. After deparaffinization and rehydration, sectioned kidneys were stained with Masson’s trichrome. For immunohistochemistry, endogenous peroxidase was blocked with 3% hydrogen peroxide, and antigen retrieval was performed with 0.01 M sodium citrate buffer (pH 6.0). Tris–EDTA buffer (pH 9.0) was used for ACOT12 staining. After blocking with normal horse serum, the sections were incubated overnight with the following primary antibodies: ACOT12 (1:1,000; AbFrontier), PLIN2 (1:1,000; Abcam), Collagen I (1:400; Southern Biotech), αSMA (1:1,000; Abcam), PPARα (1:100; Santa Cruz) and ACBD5 (1:100; Novus). After incubation with peroxidase-conjugated secondary antibodies for 1 h, the antigen was detected via ImmPACT DAB Substrate (Vector Laboratories). Images were acquired via EVOS FL Auto and quantified via Image-Pro Plus 4.5 (Cybernetics) or ImageJ software (ImageJ, National Institutes of Health; https://imagej.nih.gov/ij/).

### mRNA sequencing analysis

For mRNA sequencing analysis, total RNA was isolated from the contralateral or UUO kidneys of *Acot12*^*+/+*^ or *Acot12*^*−/−*^ mice using RNAiso Plus (Takara). The RNA quality was verified via a Bioanalyzer 2100 system (Agilent), a cDNA library was constructed using the NEBNext Ultra II Directional RNA-Seq kit (NEB) and the library was sequenced via a NovaSeq 6000 instrument (Illumina). Trimming, mapping, normalization and initial analyses were performed by E-Biogen, Inc. Further analysis was performed via ExDEGA (E-Biogen) and the Database for Annotation, Visualization and Integrated Discovery (DAVID)^21^. Networks were analyzed via the Search Tool for the Retrieval of Interacting Genes/Proteins (STRING; https://string-db.org).

### qRT–PCR

Total RNA was isolated using TRIzol reagent (Invitrogen) or RNAiso Plus (Takara), according to the manufacturer’s instructions. cDNA was synthesized via a high-capacity cDNA reverse transcription kit (Applied Biosystems) or 5× RT Master Mix (Takara). Quantitative PCR with reverse transcription (qRT–PCR) was performed using SYBR Green PCR Master Mix (Applied Biosystems) or AMPIGENE qPCR Green Mix Hi-ROX (Enzo) on an Applied Biosystems 7300 or ABI StepOnePlus instrument (Applied Biosystems). The relative expression level of each gene was normalized to the 18S rRNA expression level. The sequences of primers used in this study are listed in Supplementary Table 1.

### Western blot analysis

Kidney proteins were extracted via RIPA buffer (Cell Signaling Technology) with 2 mM phenylmethylsulfonyl fluoride, separated via sodium dodecyl sulfate‒polyacrylamide gel electrophoresis and transferred to polyvinylidene difluoride or nitrocellulose membranes (GE Healthcare BioSciences). The membranes were blocked with 5% skimmed milk in TBS–Tween 20 and incubated with the following primary antibodies: ACOT12 (1:2,000; AbFrontier), PPARα (1ː1,000; Santa Cruz) and β-actin (1:3,000; Sigma). The blots were developed with horseradish peroxidase-conjugated secondary antibodies and detected via an enhanced chemiluminescence method. The bands were quantified via ImageJ software and normalized to β-actin.

### Plasma analysis via enzyme-linked immunosorbent assay

Blood samples were collected via heparinized syringes, and the plasma was collected via centrifugation at 3,000 rpm for 20 min at 4 °C. BUN (Arbor Assays), plasma cystatin C (R&D Systems) and plasma neutrophil gelatinase-associated lipocalin (NGAL; Immunology Consultant Laboratory) were measured according to the manufacturer’s instructions.

### Statistical analyses

All the results are expressed as the means ± s.e.m. of at least three independent experiments. Representative experiments and images are shown unless otherwise stated. Two-tailed Student’s *t*-tests were used to compare two groups. One-way or two-way analysis of variance was used to assess the differences between multiple groups, followed by Fisher’s least significant difference test. Statistical tests with *P* < 0.05 were considered statistically significant.

## Results

### ACOT12 is a key factor linking lipid metabolism to kidney fibrosis

**Fig. 1:**
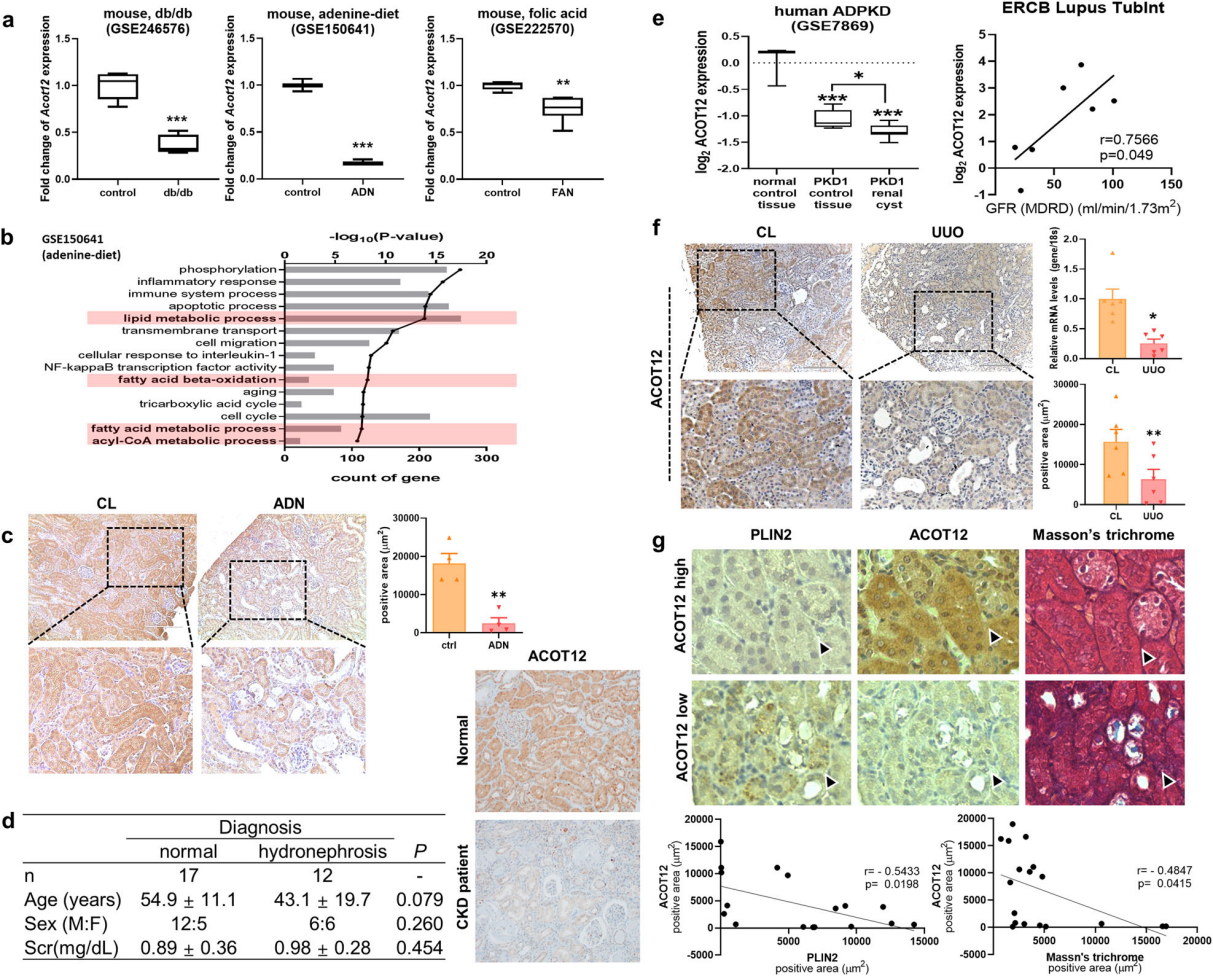
The expression level of Acot12 in a CKD model. **a** In silico analysis of *Acot12* expression levels in GSE246576 (db/db mice), GSE150641 (adenine diet-fed mice; ADN, adenine diet-induced nephropathy) and GSE222570 (folic acid-injected mice; FAN, folic acid-induced nephropathy). **b** Gene Ontology enrichment analysis of differentially expressed genes in the GSE150641 dataset (adenine diet-induced CKD mice). **c** Immunohistochemical staining of ACOT12 in adenine diet-fed control (CL) and UUO kidneys. **d** Characteristics of patients who were diagnosed with chronic kidney obstruction. Immunohistochemical staining of ACOT12 in kidney fibrosis samples from patients. **e** In silico analysis of *Acot12* expression levels in the GSE7869 dataset (human ADPKD, autosomal dominant polycystic kidney disease) and the correlation between Acot12 expression and the glomerular filtration rate calculated using the modification of diet in renal disease (MDRD) equation in the tubulointerstitium of ERCB lupus patients. The positive area was determined and is represented in a bar graph. Scale bar, 200 μm (*n* = 4). **f** Immunohistochemical staining and mRNA expression levels of ACOT12 in UUO kidneys (*n* = 6). The area positive for ACOT12 immunostaining was determined and is represented in a bar graph. Scale bar, 200 μm. The expression level of *Acot12* in UUO mice was analyzed by RT‒PCR (*n* = 6). **g** Representative images of PLIN2 and ACOT12 immunostaining and Masson’s trichrome staining according to the intensity of ACOT12 staining. Pearson correlations between the positive areas of ACOT12 and PLIN2 staining or Masson’s trichrome staining are shown. **P < 0.01 and ***P < 0.001.

UUO, which mimics chronic obstructive nephropathy in humans, results in kidney fibrosis as well as lipid and extracellular matrix accumulation^22^ (Supplementary Fig. 1a). Perilipin 2 (PLIN2), which coats intracellular lipid droplets, was observed as a marker of lipid metabolism activation^23^. In addition, treatment with losartan, an angiotensin II type 1a receptor antagonist, attenuated fibrosis^24^ and decreased lipid deposition in UUO-induced mice (Supplementary Fig. 1a). These results suggest that dysregulated lipid metabolism is closely associated with the development and progression of kidney fibrosis. Next, genome-wide transcript levels in the UUO model were analyzed to identify the key player responsible for lipid accumulation and fibrosis (Supplementary Fig. 1b). Gene Ontology analysis revealed that three metabolic processes, the FA metabolic process (*n* = 26), lipid metabolic process (*n* = 44) and acyl-CoA metabolic process (*n* = 12), were significantly altered in UUO kidneys compared with control kidneys (Supplementary Fig. 1c). Moreover, acyl-CoA synthetase medium-chain family members (*Acsm*) 1/2/3/5, *Acot 11/12*, enoyl-CoA hydratase and 3-hydroxyacyl CoA dehydrogenase (*Ehhadh*) were common genes in all three groups. Acyl-CoA synthetase medium-chain (ACSM)s generate acyl-CoA from free FAs and CoA, whereas ACOTs hydrolyze acyl-CoA to FAs and CoA. EHHADH is a peroxisomal β-oxidation enzyme. The protein–protein interaction network constructed via the STRING database suggested that ACOT12 is a key player in lipid accumulation and fibrosis (Supplementary Fig. 1d). The transcriptional and translational levels of ACOT12 were significantly lower in UUO kidneys than in control kidneys (Supplementary Fig. 1e). Furthermore, losartan treatment was associated with an increase in ACOT12 expression, which correlated with reduced kidney injury (Supplementary Fig. 1f). This observation is consistent with various models, including diabetes (GSE246576), an adenine-rich diet (GSE150641) and folic acid exposure (GSE222570), all of which are associated with decreased levels of ACOT12 expression (Fig. 1a). The adenine-rich diet models revealed common lipid-related pathways that matched those found in our UUO mRNA sequencing data, highlighting the consistent enrichment of signaling pathways across these various conditions (Fig. 1b). Diminished expression of ACOT12 in kidneys subjected to an adenine-rich diet was further confirmed through immunohistochemistry (Fig. 1c). In human kidney biopsies, we noted a significant reduction in ACOT12 compared with that in normal kidneys (Fig. 1d). In silico analysis using Gene Expression Omnibus (GEO) (GSE7869) confirmed that ACOT12 expression was lower in patients with autosomal dominant polycystic kidney disease (ADPKD) than in normal patients (Fig. 1e). Analysis of the tubulointerstitial transcriptome from European Renal cDNA Bank (ERCB) patients with CKD identified in the NephroSeq v5 online database revealed a significant positive correlation between ACOT12 and the estimated glomerular filtration rate (eGFR). Consistent with these findings, we also observed a significant decrease in ACOT12 in UUO kidneys compared with that in contralateral kidneys (Fig. 1f). A negative correlation between PLIN2 expression/fibrosis and ACOT12 expression was observed not only via immunohistochemistry images, but also via Pearson correlation analysis of those images (Fig. 1g).

### *Acot12*^*−/−*^ mice develop age-related renal fibrosis

**Fig. 2:**
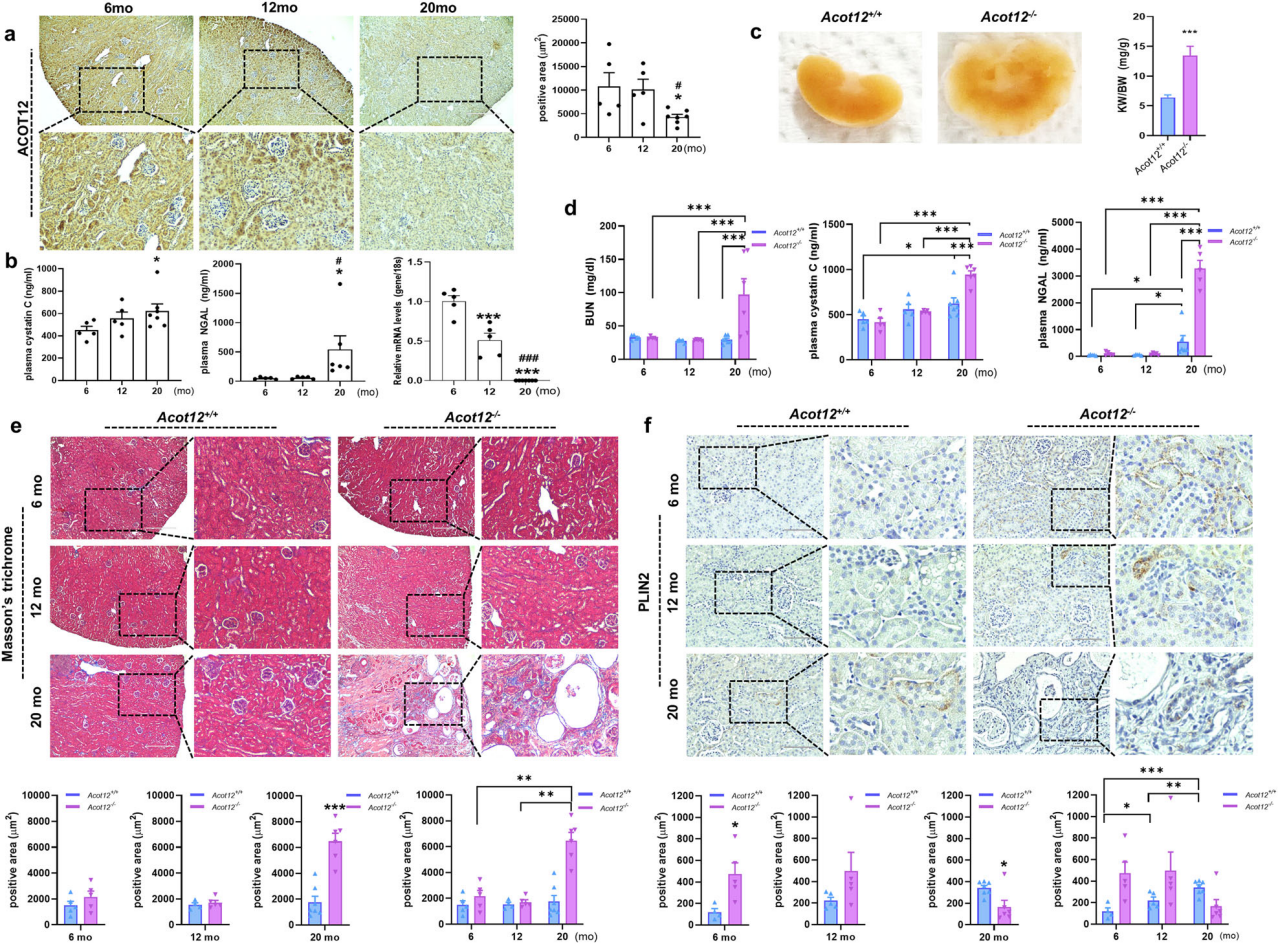
Association of ACOT12 deficiency with aging-dependent loss of kidney function. **a** Immunohistochemical staining and mRNA expression levels of Acot12 in 6-, 12- and 20-month-old kidneys (*n* = 5–7 in each group). 6 mo, 6-month-old; 12 mo, 12-month-old; 20 mo, 20-month-old. Scale bar, 400 μm. **b** Plasma levels of cystatin C and NGAL in 6-, 12- and 20-month-old ACOT12 mice (*n* = 5‒7 per group). **c** Representative kidney and KW to BW ratios of 20-month-old *Acot12*^*+/+*^ or *Acot12*^*−/−*^ mice. **d** The plasma levels of urea nitrogen, cystatin C and NGAL in 6-, 12- and 20-month-old *Acot12*^*+/+*^ and *Acot12*^*−/−*^ mice were analyzed (*n* = 5‒7 per group). **e** Masson’s trichrome staining of 6-, 12- and 20-month-old *Acot12*^*+/+*^ and *Acot12*^*−/−*^kidneys. The positive area was determined and the data are presented as a bar graph. Scale bar, 200 μm. **f** Immunohistochemical staining of PLIN2 in 6-, 12- and 20-month-old *Acot12*^*+/+*^ and *Acot12*^*−/−*^ mice. The positive area was determined and the data are presented as a bar graph. Scale bar, 100 μm. *P < 0.05, **P < 0.01, ***P < 0.001 and ^#^*P* < 0.05 per 12-month-old kidney.

Here, we observed an age-dependent loss of ACOT12 expression in the renal tubules (Fig. 2a). In the kidneys of 20-month-old *Acot12*^*+/+*^ mice, the expression level of ACOT12 dramatically decreased, with increased kidney dysfunction and injury observed via plasma cystatin C and NGAL analysis (Fig. 2b). *Acot12* KO (*Acot12*^*−/−*^) mice previously generated via RGEN-induced mutation of the *Acot12* gene (Supplementary Fig. 2) were used to understand the role of ACOT12 in kidney disease pathogenesis. In 20-month-old *Acot12*^*−/−*^ mice, the average kidney size was markedly larger than that in *Acot12*^*+/+*^ mice (Fig. 2c and Supplementary Fig. 3a). Significant increases in three types of kidney dysfunction and injury markers in the plasma, namely, BUN, cystatin C and NGAL, were also observed (Fig. 2d). Masson’s trichrome staining revealed a significant increase in the number of polycysts in the kidneys of 20-month-old *Acot12*^*−/−*^ mice compared with *Acot12*^*+/+*^ mice (Fig. 2e). Age-dependent increases in kidney fibrosis (Fig. 2e and Supplementary Fig. 3b) were observed, with the most significant increase observed in 20-month-old *Acot12*^*−/−*^ kidneys. Significant lipid accumulation was observed in the kidneys of 6- and 12-month-old *Acot12*^*−/−*^ mice compared with those of *Acot12*^*+/+*^ mice (Fig. 2f). This early lipid deposition suggests that dysregulated lipid metabolism due to ACOT12 deficiency may contribute to the initiation and progression of kidney fibrosis, highlighting the role of lipid accumulation as an early pathogenic event in kidney disease.

### ACOT12 restoration attenuates lipid accumulation and kidney fibrosis

**Fig. 3:**
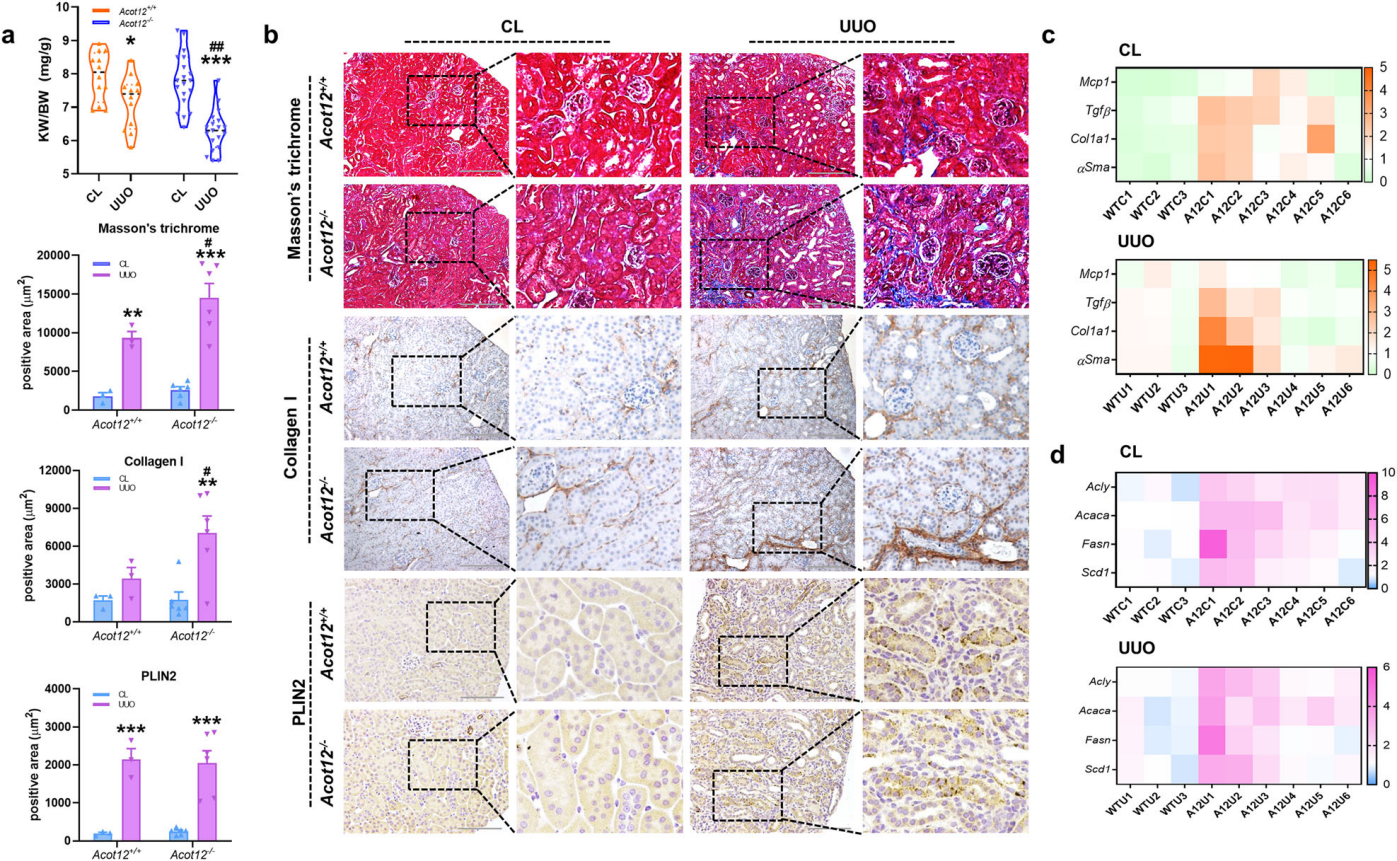
ACOT12 deficiency exacerbates lipid accumulation and kidney fibrosis. **a** The KW to BW ratio in UUO-induced *Acot12*^*+/+*^ and *Acot12*^*−/−*^ mice (*n* = 12–19 per group). **b** Masson’s trichrome and immunohistochemical staining of collagen I and PLIN2 in *Acot12*^*+/+*^ UUO kidneys and UUO *Acot12*^*−/−*^ UUO kidneys (*n* = 3‒6 per group). The positive area was determined and the data are presented as a bar graph. Scale bar for images of Masson’s trichrome staining, 200 μm. Scale bar for images of PLIN2 staining, 100 μm. **c** The expression levels of inflammatory (*Mcp1* and *Tgfβ*) and fibrotic (*Col1a1* and *αSma*) genes in *Acot12*^*+/+*^ UUO kidneys and *Acot12*^*−/−*^ UUO kidneys were analyzed by RT‒PCR and are presented as a heat map (*n* = 3–6 per group). WTC, wild-type control; WTU, wild-type UUO; ATC, *Acot12*^-/-^ control; ATU, *Acot12*^-/-^ UUO. **d** The expression levels of lipid synthesis genes (*Acly*, *Acaca*, *Fasn* and *Scd1*) in *Acot12*^*+/+*^ and *Acot12*^*−/−*^ UUO kidneys were analyzed via RT‒PCR and are represented as heat maps (*n* = 3‒6 per group). *P < 0.05, **P < 0.01 and ***P < 0.001 per CL. ^#^*P* < 0.05, ^##^*P* < 0.01 and ^###^*P* < 0.001 per *Acot12*^*+/+*^ UUO.

To fully understand the role of ACOT12 in the pathogenesis of kidney disease, *Acot12*^*−/−*^ mice subjected to UUO surgery were used. The kidney weight to body weight ratio (KW/BW) was significantly lower in *Acot12*^*−/−*^ kidneys than in *Acot12*^*+/+*^ kidneys (Fig. 3a). Kidney fibrotic lesions, as assessed by Masson’s trichrome and type I collagen staining, were significantly greater in *Acot12*^*−/−*^ kidneys than in *Acot12*^*+/+*^ kidneys (Fig. 3b). The expression levels of genes associated with inflammation, such as *Mcp1*, or fibrosis, such as *Tgfβ, Col1a1* and *αsma*, were increased in *Acot12*^*−/−*^ kidneys, with the most significant increase observed in UUO-induced *Acot12*^*−/−*^ kidneys (Fig. 3c). The expression levels of genes involved in lipid synthesis, such as *Acly, Acaca, Fasn* and *Scd1*, were also increased in *Acot12*^*−/−*^ kidneys and UUO-induced *Acot12*^*−/−*^ kidneys (Fig. 3d). However, PLIN2 staining revealed no difference in lipid accumulation between *Acot12*^*−/−*^ and *Acot12*^*+/+*^ kidneys.

**Fig. 4:**
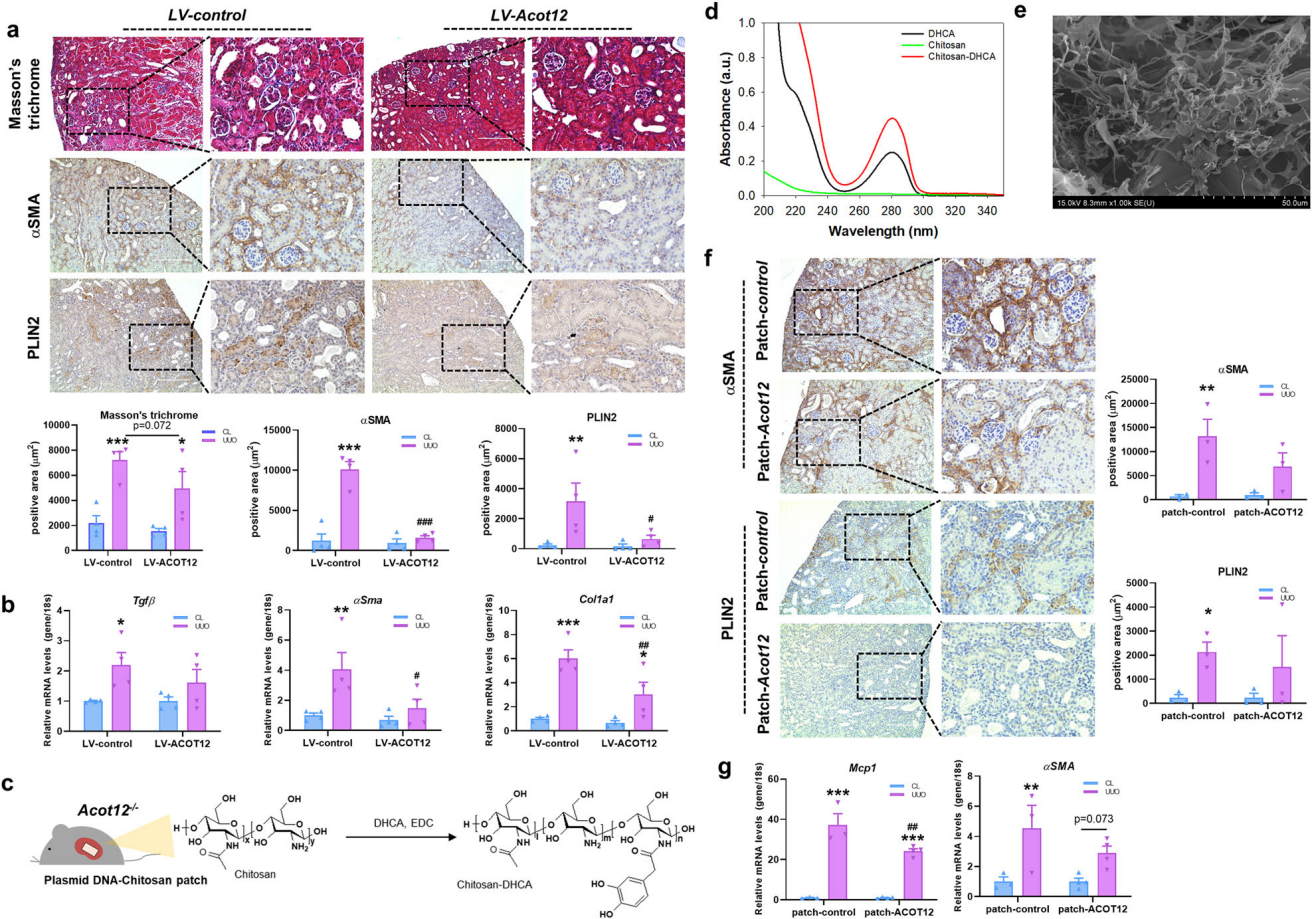
Restoration of ACOT12 ameliorates lipid accumulation and kidney fibrosis in *Acot12*^*−/−*^ UUO kidneys. **a** Masson’s trichrome and immunohistochemical staining of αSMA and PLIN2 in *Acot12*^*−/−*^ UUO kidneys with or without parenchymal injection of lentivirus containing the *Acot12* construct (LV*-Acot12*) (*n* = 4 per group). A control lentivirus (LV*-control*) was used as a negative control. The positive area was determined and the data are presented as a bar graph. Scale bar, 200 μm. **b** The expression levels of fibrotic genes in *Acot12*^*−/−*^ UUO kidneys with or without parenchymal injection of LV*-Acot12* (*n* = 4 per group). **c** Synthesis and chemical structure of CHI–DHCA. **d** UV‒Vis spectra of the DHCA monomer, chitosan and CHI–DHCA. **e** Scanning electron microscope image of the CHI–DHCA patch. **f** Immunohistochemical staining of αSMA and PLIN2 in *Acot12*^*−/−*^ UUO kidneys with or without a chitosan patch conjugated with the *Acot12* expression vector (*n* = 3 per group). The positive area was determined and is represented in a bar graph. Scale bar, 200 μm. **g** The expression levels of fibrotic and inflammatory genes in *Acot12*^*−/−*^ UUO kidneys with or without chitosan patch-conjugation with the *Acot12* expression vector (*n* = 3 per group). *P < 0.05, **P < 0.01 and ***P < 0.001 per control CL. ^#^*P* < 0.05, ^##^*P* < 0.01 and ^###^*P* < 0.001 per control UUO.

Restoration of ACOT12 expression using a lentivirus (*LV-Acot12*) via parenchymal injection decreased kidney fibrosis and lipid accumulation (Fig. 4a and Supplementary Fig. 4a) as well as the expression levels of fibrosis-related genes by day 7 post-UUO (Fig. 4b). Additionally, on day 3 post-UUO, kidney fibrosis was reduced and ACOT12 was restored (Supplementary Fig. 4b,c). We also delivered *Acot12* via a chitosan patch. To prepare the DNA-loaded adhesive patches, CHI–DHCA was first synthesized via standard carbodiimide chemistry. As shown in Fig. 4c, the primary amines of chitosan could react with the carboxylic acid groups of DHCA, resulting in the synthesis of CHI–DHCA. The DHCA conjugation of CHI–DHCA was confirmed by UV‒Vis spectroscopy. As previously reported, the catechol groups of DHCA exhibit an absorption maximum at a wavelength of 280 nm (ref. ^25^). The typical absorption of catechol groups at 280 nm was detected in both DHCA and CHI–DHCA (Fig. 4d). The degree of catechol substitution of CHI–DHCA was 8.7%. Next, the CHI–DHCA patches were prepared via a simple freeze-drying method. CHI–DHCA was subsequently dissolved in ddH_2_O and freeze dried. Uniform sponge-like structures were found in the CHI–DHCA patches (Fig. 4e). The application of the chitosan patch with the *Acot12* expression vector (patch-ACOT12) to the kidney significantly reduced kidney fibrosis (Fig. 4f and Supplementary Fig. 4d,e). Furthermore, the levels of inflammatory and fibrotic genes were also simultaneously lower in UUO kidneys attached with patch-ACOT12 than in control kidneys (patch-control) (Fig. 4g).

### ACOT12 deficiency exacerbates kidney fibrosis independently of PPARα

**Fig. 5:**
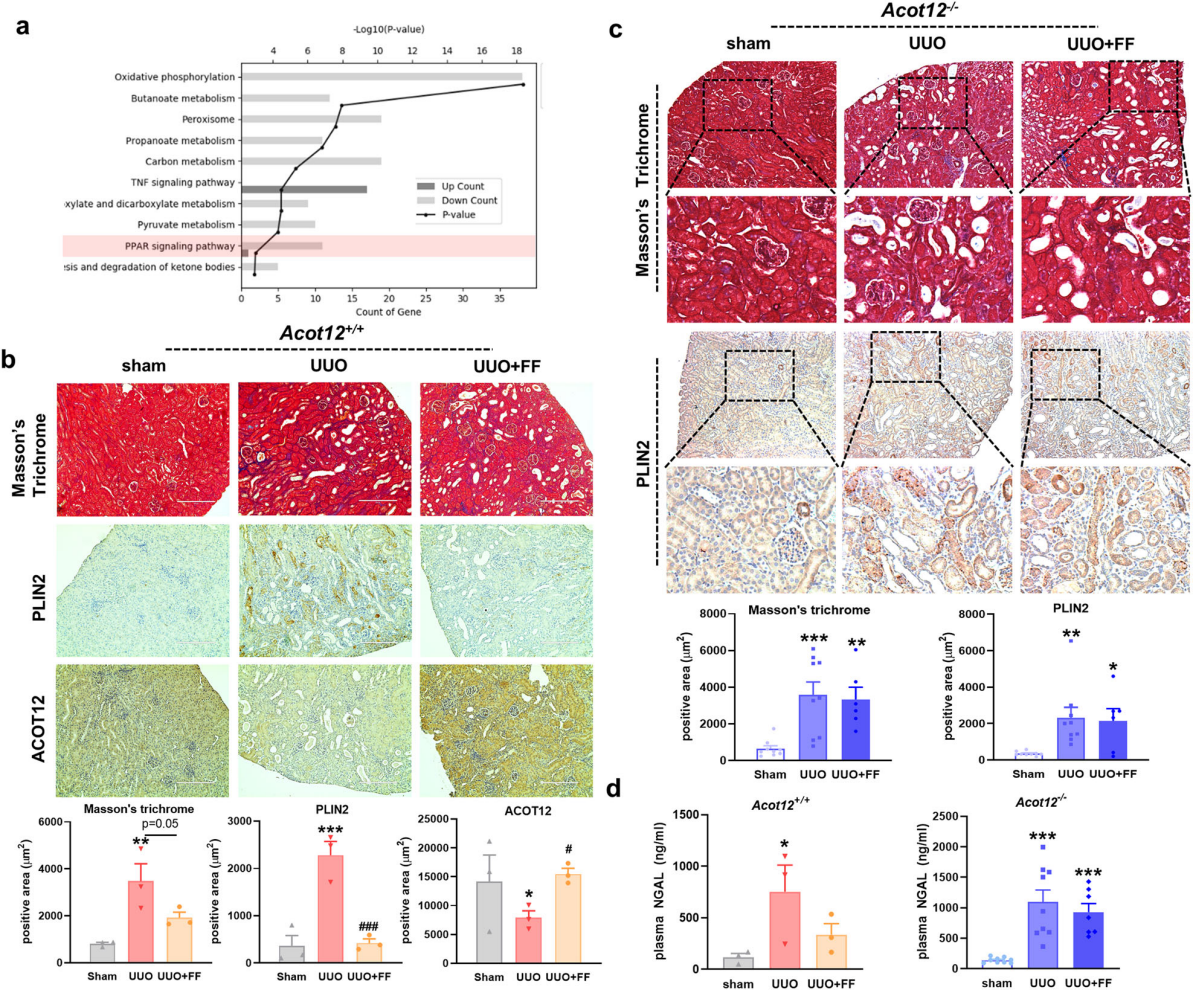
ACOT12 deficiency may stimulate kidney fibrosis independently of PPARα. **a** Gene Ontology enrichment analysis of differentially expressed genes in *Acot12*^*−/−*^ UUO kidneys compared to *Acot12*^+/+^ UUO kidneys. **b** Masson’s trichrome and immunohistochemical staining of PLIN2 and ACOT12 in *Acot12*^*+/+*^ UUO kidneys with or without oral administration of fenofibrate (FF) (*n* = 3 per group). The positive area was determined and the data are presented as a bar graph. Scale bar, 200 μm. **c** Masson’s trichrome and immunohistochemical staining of PLIN2 in *Acot12*^*−/−*^ UUO kidneys with or without oral administration of fenofibrate (*n* = 6‒9 per group). The positive area was determined and the data are presented as a bar graph. Scale bar, 200 μm. **d** Plasma NGAL levels in *Acot12*^*+/+*^ and *Acot12*^*−/−*^ UUO kidneys with or without oral administration of fenofibrate (*n* = 3–9 per group). *P < 0.05, **P < 0.01 and ***P < 0.001 per sham. ^#^*P* < 0.05 and ^###^*P* < 0.001 per UUO.

**Fig. 6:**
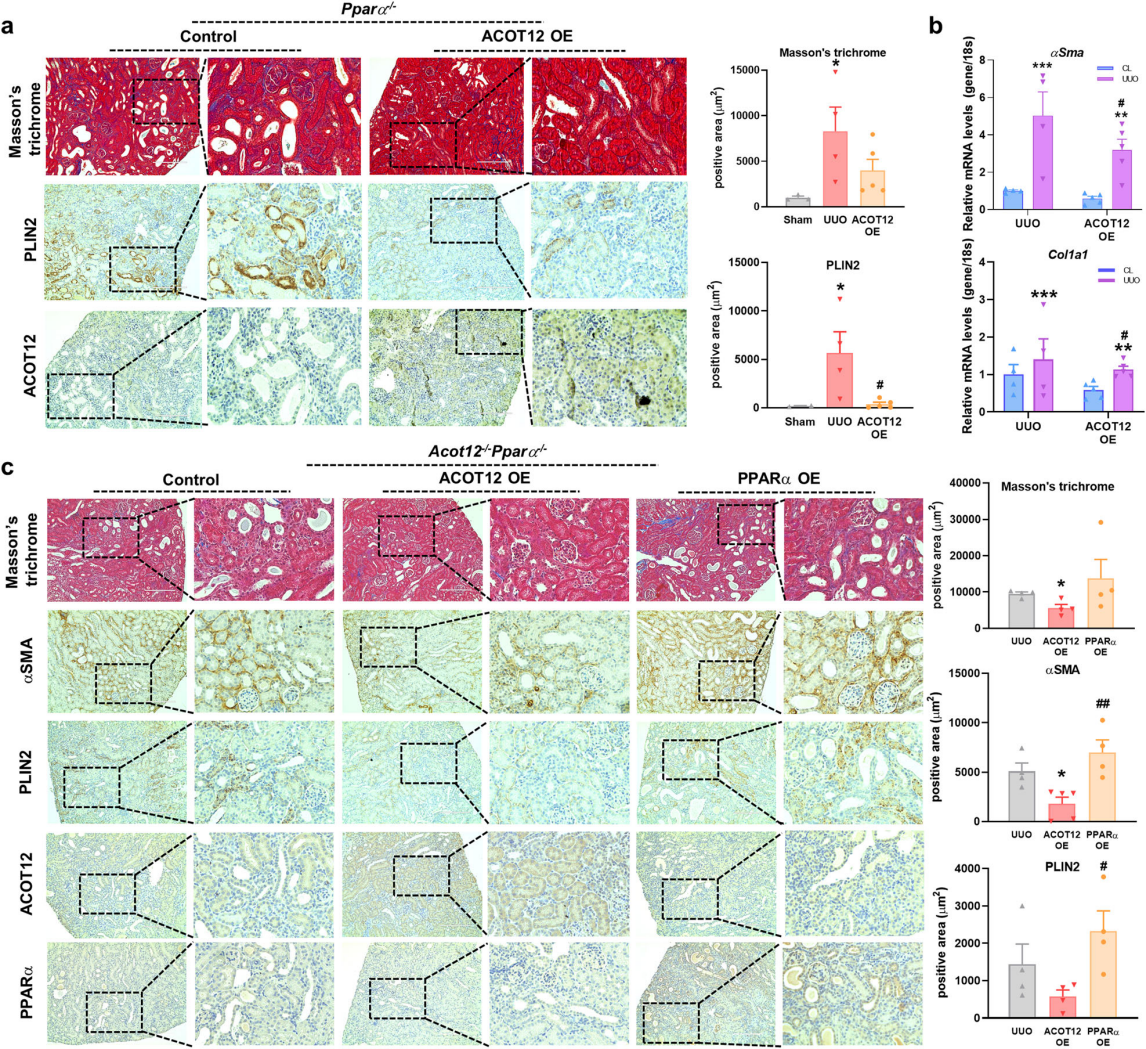
Restoration of Acot12 into *Acot12*^*−/−*^*Pparα*^*−/−*^ kidneys reduces kidney fibrosis but restoration of PPARα does not. **a** Masson’s trichrome and immunohistochemical staining of PLIN2 and ACOT12 in *Pparα*^*−/−*^ UUO kidneys with or without ACOT12 overexpression (ACOT12OE) (*n* = 3–5 per group). The positive area was determined and the data are presented as a bar graph. Scale bar, 200 μm. **b** The expression levels of fibrotic genes in *Pparα*^*−/−*^ UUO kidneys with or without ACOT12 introduction were analyzed by RT‒PCR (*n* = 3‒5 per group). **c** Masson’s trichrome and immunohistochemical staining of αSMA, PLIN2, ACOT12 and PPARα in *Acot12*^*−/−*^*Pparα*^*−/−*^ UUO kidneys without or with ACOT12 or PPARα overexpression (*n* = 4 per group). The positive area was determined and the data are presented as a bar graph. Scale bar, 200 μm. *P < 0.05, **P < 0.01 and ***P < 0.001 per sham or control CL. ^#^*P* < 0.05 and ^##^*P* < 0.01 per control or ACOT12OE UUO.

Previous research has demonstrated that PPAR plays a key role in protecting the kidney from CKD as well as acute injury^5^. As shown in Fig. 5a, the PPAR pathway emerged as a critical biological pathway that was significantly altered in the pathogenesis of kidney fibrosis. Notably, ACOT12 expression seems to be regulated by PPARα due to the presence of a predicted PPAR-binding motif. To further test whether the activation of PPARα could overcome kidney fibrosis induced by ACOT12 deficiency, fenofibrate, a known PPARα agonist, was used. In UUO-induced *Acot12*^*+/+*^ mice, fenofibrate treatment significantly reduced lipid accumulation and kidney fibrosis and increased ACOT12 expression (Fig. 5b). However, fenofibrate treatment did not restore kidney fibrosis in UUO-induced *Acot12*^*−/−*^ mice, although fenofibrate activated PPARα (Fig. 5c and Supplementary Fig. 5a). Consistent with this finding, a significant decrease in plasma NGAL levels was observed in the fenofibrate-treated *Acot12*^*+/+*^ mice, whereas plasma NGAL levels were not altered in the fenofibrate-treated *Acot12*^*−/−*^ mice (Fig. 5d). These data suggest that kidney fibrosis induced by ACOT12 deficiency is caused by a signaling pathway independent of PPARα signaling. To verify this, we used *Pparα*^*−/−*^ mice to generate *Acot12* and *Pparα* double-KO (*Acot12*^*−/−*^ *Pparα*^*−/−*^) mice (Supplementary Fig. 2). Lentiviral introduction of ACOT12 into UUO-induced *Pparα*^*−/−*^ mice via parenchymal injection significantly reduced lipid accumulation and kidney fibrosis (Fig. 6a), as did the expression levels of fibrosis genes (Fig. 6b). Moreover, the restoration of ACOT12 in *Acot12*^*−/−*^ *Pparα*^*−/−*^ mice significantly reduced lipid accumulation and kidney fibrosis (Fig. 6c). Surprisingly, however, restoring PPARα in *Acot12*^*−/−*^ *Pparα*^*−/−*^ mice exacerbated lipid accumulation and kidney fibrosis.

### ACOT12 deficiency inhibits pexophagy via the downregulation of ACBD5

**Fig. 7:**
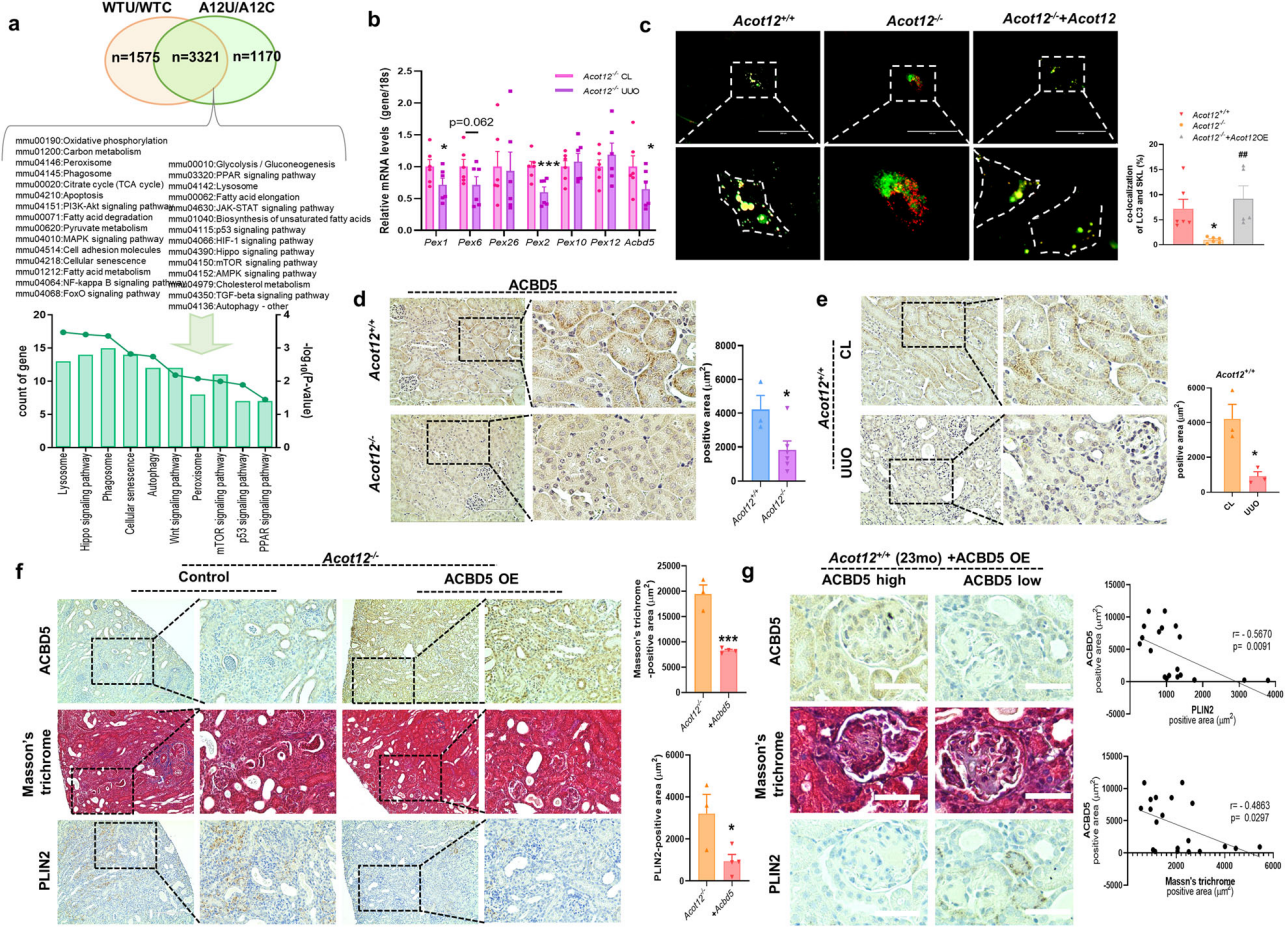
ACOT12 deficiency may inhibit pexophagy via the downregulation of ACBD5. **a** A Venn diagram of differentially expressed genes in *Acot12*^*+/+*^ UUO or *Acot12*^*−/−*^ UUO kidneys and analysis of Kyoto Encyclopedia of Genes and Genomes pathways enriched in *Acot12*^*−/−*^ UUO kidneys. **b** The expression levels of pexophagy-related genes in *Acot12*^*−/−*^ UUO kidneys were analyzed via RT‒PCR (*n* = 6 per group). **c** Representative image of colocalization of GFP-LC3 and RFP-SKL in primary kidney tubular epithelial cells isolated from *Acot12*^*+/+*^ and *Acot12*^*−/−*^ kidneys or in ACOT12-transfected *Acot12*^*−/−*^ primary kidney tubular epithelial cells. The colocalization of LC3 and SKL was quantified and the results are presented as a graph. Magnification of the images is shown in areas demarcated by the white box. Scale bar, 100 μm. Immunohistochemical staining of ACBD5 in *Acot12*^*+/+*^ or *Acot12*^*−/−*^ kidneys (**d**) and in *Acot12*^+/+^ kidneys with or without UUO surgery (**e**). The positive area was determined and the data are presented as a bar graph (*n* = 3–6 per group). Scale bar, 100 μm. **f** Masson’s trichrome and immunohistochemical staining of PLIN2 and ACBD5 in *Acot12*^*−/−*^ UUO kidneys with or without ACBD5 overexpression (ACBD5 OE) (*n* = 3–4 per group). The positive area was determined and the data are presented as a bar graph. Scale bar, 200 μm. **g** ACBD5 was recovered in 23-month-old *Acot12*^*+/+*^ kidneys. Images of PLIN2 and ACBD5 immunohistochemical staining or Masson’s trichrome staining and correlations between ACBD5 and PLIN2 or ACBD5 and Masson’s trichrome staining, scale bar, 50 μm. *P < 0.05 and ***P < 0.001.

To investigate the underlying mechanism involved in kidney fibrosis induced by ACOT12 deficiency, we extracted genes differentially expressed in *Acot12*^*−/−*^ UUO kidneys or commonly overlapping genes and analyzed them via the DAVID bioinformatics resource^26^ (Fig. 7a). The lysosome, peroxisome, phagosome, autophagy, cellular senescence, mTOR and p53 signaling pathways were enriched in genes differentially expressed in *Acot12*^*−/−*^ UUO kidneys. Among these, significant attention was given to peroxisomal biogenesis factor (PEX) genes, such as Pex1 and Pex2, which were found to be downregulated, indicating disrupted peroxisome biosynthesis in the absence of ACOT12 (Fig. 7b). In addition to a reduction in the expression of peroxisomal biosynthesis genes (Fig. 7b and Supplementary Fig. 5b), the expression of acyl-CoA-binding domain containing 5 (*Acbd5*), a gene encoding the peroxisome receptor for pexophagy, was also significantly decreased in *Acot12*^*−/−*^ UUO kidneys. Given the critical role of ACBD5 in facilitating the interaction between peroxisomes and autophagosomes, its downregulation suggests a potential mechanism through which impaired pexophagy contributes to the fibrotic phenotype observed in these kidneys. The colocalization of peroxisomes with the autophagosome marker LC3 was more prominent in *Acot12*^*+/+*^ primary murine tubular epithelial cells than in *Acot12*^*−/−*^ cells, indicating the activation of pexophagy (Fig. 7c and Supplementary Fig. 5c). However, in *Acot12*^*−/−*^ primary murine tubular epithelial cells, a significantly lower colocalization level was observed, which was rescued by the restoration of *Acot12*. Immunohistochemical staining revealed that ACBD5 expression was significantly lower in *Acot12*^*−/−*^ kidneys than in *Acot12*^*+/+*^ kidneys and in *Acot12*^*+/+*^ UUO kidneys than in contralateral kidneys (Fig. 7d,e). The decreased level of ACBD5 in *Acot12*^*+/+*^ UUO kidneys was reversed by treatment with losartan and fenofibrate (Supplementary Fig. 6a,b), which was previously shown to induce ACOT12 expression. However, fenofibrate treatment did not lead to an increase in ACBD5 expression in *Acot12*^*−/−*^ UUO kidneys. Consistent with ACOT12 expression, an age-dependent loss of ACBD5 expression was observed (Supplementary Fig. 6c), suggesting that the fibrosis induced by ACOT12 may be linked to pexophagy. The overexpression of ACBD5 in *Acot12*^*−/−*^ UUO kidneys also ameliorated kidney fibrosis and lipid accumulation (Fig. 7f). Although the introduction of ACOT12 or ACBD5 into 23-month-old *Acot12*^*+/+*^ kidneys did not achieve as high a level of overexpression as that observed after earlier injections, the areas where ACOT12 or ACBD5 was overexpressed presented notable improvements in kidney fibrosis and lipid accumulation (Fig. 7g and Supplementary Fig. 7).

## Discussion

Accumulating evidence has revealed that defective FAO in the kidneys can induce kidney fibrosis^11,27^. Tubular epithelial cells in the kidney use FA instead of glucose as the major energy source for ATP generation, and the importance of mitochondrial β-oxidation has been suggested^28–30^. The downregulation of the CPT family proteins CPT1 and CPT2, which are involved in transporting FAs into the mitochondria, induces FA accumulation and contributes to CKD and kidney fibrosis^12,31^. In this study, we found that ACOT12 KO exacerbated lipid accumulation and kidney fibrosis in kidney tubules.

ACOT12, which is found in the cytosol and peroxisomes and regulates acetyl-CoA metabolism, is highly expressed in the liver, kidney and intestine^32^. ACOT12 deficiency is known to increase de novo lipogenesis (DNL) and leads to the development of nonalcoholic fatty liver disease^19,33^. In hepatocellular carcinoma, the accumulation of acetyl-CoA induced by ACOT12 deficiency increases the acetylation of TWIST2 and stimulates epithelial-mesenchymal transition (EMT)^34^. In addition, the activation of acetyl-CoA synthetases increases DNL by stimulating the acetylation of acetyl-CoA carboxylase and FA synthase in hepatocellular carcinoma under hypoxia^35^. These data suggest various biological and pathological roles for ACOT12. However, the pathophysiological role and mechanism of action of ACOT12 in kidney diseases remain unknown. Here, we found that ACOT12 deficiency significantly exacerbated kidney fibrosis in both aged and UUO-induced mice. Severe polycystic kidney injury and early lipid deposition in aged ACOT12-deficient mice indicate that the dysregulation of lipid metabolism is a critical factor in the aging process of the kidneys^6^. A recent study demonstrated that deficiency of PPARα stimulates DNL by modulating ACOT12 (ref. ^19^). Moreover, the administration of MHY2013, a PPARα/β agonist, reduced accumulation and alleviated age-associated kidney fibrosis^36^. As defective lipid metabolism, such as impaired FAO signaling, has recently been implicated in kidney fibrosis, treatment with a PPARα agonist or overexpression of PPARα could be expected to exert an antifibrotic effect in a kidney fibrosis model. Here, we observed that exposure of UUO kidneys to fenofibrate reduces lipid accumulation and increases ACOT12 expression in kidney tubules. However, fenofibrate introduction to *Acot12*^*−/−*^ UUO kidneys did not ameliorate lipid accumulation or reverse fibrosis. Interestingly, restoring PPARα in *Pparα*^*−/−*^*Acot12*^*−/−*^ kidneys exacerbated lipid accumulation and kidney fibrosis, suggesting an important role of ACOT12 in kidney fibrosis independent of PPARα.

Several studies have reported the relationship between autophagy and kidney fibrosis^37–39^. Autophagy deficiency increases the sensitivity of kidneys to damage, leading to the accumulation of damaged mitochondria, impaired kidney function and severe kidney fibrosis^40^. Moreover, the accumulation of cytosolic acetyl-CoA, potentially resulting from ACOT12 deficiency, is known to lead to the activation of acetyltransferase EP300 and mTORC1, and results in the inhibitory acetylation of autophagy-related proteins, leading to the suppression of autophagy^41,42^. In this study, we found that pexophagy, a type of macroautophagy that selectively degrades peroxisomes, was decreased in the kidney tubules of *Acot12*^*−/−*^ UUO kidneys. The accumulation of defective peroxisomes or failure to remove defective peroxisomes due to dysfunctional pexophagy could alter cellular redox homeostasis and/or impair peroxisome regeneration^43,44^. Defective pexophagy in the liver reportedly increases oxidative stress and liver injury and contributes to the development of liver fibrosis^45^. The accumulation of impaired peroxisomes caused by pexophagy inhibition results in redox imbalance and kidney damage in the acute kidney injury model^46^. It has also been reported that signaling pathways involved in fibrosis, such as the TGFβ signaling pathway, can affect peroxisome proliferation/dynamics^47^. In this study, we observed decreased levels of peroxisomal biogenic factors in *Acot12*^*−/−*^ UUO kidneys. Moreover, we detected significantly decreased expression levels of ACBD5, which is essential for pexophagy and is required for the delivery of peroxisomes to lysosomes in *Acot12*^*−/−*^ UUO kidneys^48,49^. These data indicate an association between pexophagy and kidney fibrosis induced by ACOT12 deficiency. However, the detailed mechanisms underlying this association and the extent of its involvement in different fibrotic conditions need to be investigated. Although an association between ACOT12 deficiency and kidney fibrosis has been identified, the precise mechanisms underlying this relationship and its prevalence across various fibrotic states require further investigation. Moreover, additional studies using conditional KO models are needed to closely examine the kidney-specific effects of ACOT12 deficiency. Considering the differences in susceptibility to kidney injury and peroxisome dysfunction between sexes, the implications of ACOT12 deficiency in females also need to be explored^50–54^.

In summary, we used transgenic mouse models to delineate the biological functions of ACOT12 in the pathogenesis of kidney diseases, particularly kidney fibrosis. Our results demonstrated the important in vivo function of ACOT12 as a key regulator of lipid metabolism and kidney fibrosis. ACOT12 deficiency exacerbates kidney fibrosis possibly through the downregulation of pexophagy.

## Data and materials availability

The authors confirm that all the data are available in the main text or the Supplementary Materials. For general comments or material requests, please contact E.-J.J. (jineunjung@wku.ac.kr). The sequencing data that support the findings of this study can be accessed under the Gene Expression accession number GSE261115.

## Supplementary information

Supplementary information.
