# Supplementary material for: ACOT12, a novel factor in the pathogenesis of kidney fibrosis, modulates ACBD5
Source: Exp Mol Med. 2025 Feb 13;57(2):478–88. doi: 10.1038/s12276-025-01406-3 (PMC11873122; doi:10.1038/s12276-025-01406-3)
Supplement: Supplementary file 1 — Supplementary information. [file 12276_2025_1406_MOESM1_ESM.pdf]

ACOT12, a novel factor in the pathogenesis of kidney fibrosis, modulates ACBD5

Ee Hyun Kim[1,5], Mi Kyung Kim[2], Mi Sun Choe[3], Ji Hyun Ryu[4], Eun Seon Pak[1], Hunjoo Ha[1]*, Eun-Jung Jin[4, 5]*

[1]Graduate School of Pharmaceutical Sciences, College of Pharmacy, Ewha Womans University, Seoul, 03760, South Korea

[2]Department of Internal Medicine, School of Medicine, Institute for Medical Science, Keimyung University, Daegu, 42601, South Korea

[3]Department of Pathology, School of Medicine, Institute for Medical Science, Keimyung University, Daegu, 42601, South Korea

[4]Department of Biomedical Materials Science, Wonkwang University, Iksan, Jeonbuk State, 54538, South Korea.

[5]Integrated Omics Institute, Wonkwang University, Iksan, Jeonbuk State, 54538, South Korea

Running head: A novel role of ACOT 12 in kidney fibrosis

*Corresponding authors

Hunjoo Ha

Address: Graduate School of Pharmaceutical Sciences, College of Pharmacy, Ewha Womans University, Seoul 03760, Republic of Korea

Telephone/Fax number: +82-2-3277-3001/+82-2-3277-2851 Email: hha@ewha.ac.kr

Eun-Jung Jin2

Address: Department of Biomedical Materials Science, Wonkwang University, Iksan, Jeonbuk State, 54538, South Korea

Telephone/Fax number: +82-063-850-6192/+82-63-850-6197 Email: jineunjung@wku.ac.kr

Running head: Role of acyl-CoA thioesterase 12 in kidney fibrosis

SUPPLEMENTARY FIGURES

Supplementary Fig.1

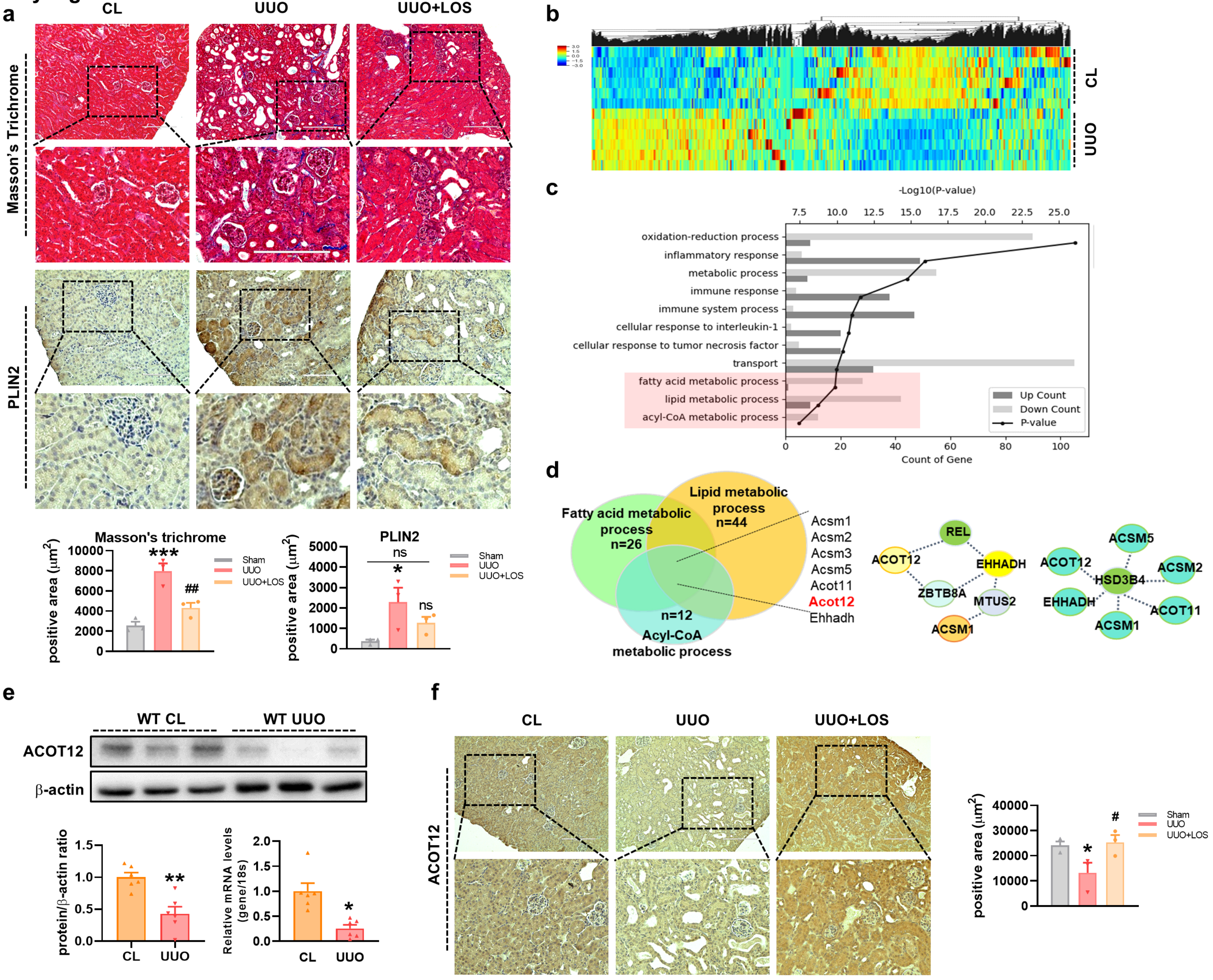

Supplementary Fig. 1. ACOT12 plays an important role in the pathogenesis of kidney diesease. a Masson's Trichrome and immunohistochemical staining images of PLIN2 in UUO kidney treated with or without Losartan (LOS). The untreated contralateral kidney (CL) was used as a control. Scale bar = 200 μm for Masson's Trichrome staining images, 100 μm for PLIN2 staining images. **b** Heatmaps illustrating hierarchical clustering of significant transcripts expressed in UUO kidneys (n=6). Colors represent the level of gene expression relative to the corresponding control level and median centered for each gene, with blue, green, and red corresponding to lower, equal, and higher expression, respectively. **c** Gene Ontology enrichment analysis of differentially expressed genes in UUO kidneys. **d** A Venn diagram and STRING analysis of three metabolic processes from gene ontology enrichment analysis. **e** mRNA and protein levels of ACOT12 in UUO kidneys. Expression levels of *Acot12* in UUO kidneys were quantified using reverse transcription-quantitative polymerase chain reaction (RT-qPCR) relative to the housekeeping gene RN18S and given as ΔΔ Ct fold change relative to the untreated control (n=6 per group). The blot was quantified with Image J software and represented as quantitative graph. β-actin was used as a loading bar graph. **f** Immunohistochemical staining of ACOT12 in UUO kidneys treated with or without LOS. Scale bar = 200 μm. * $P < 0.05$; ** $P < 0.01$; *** $P < 0.001$ per CL # $P < 0.05$; ## $P < 0.01$ per UUO.

Supplementary Fig. 2

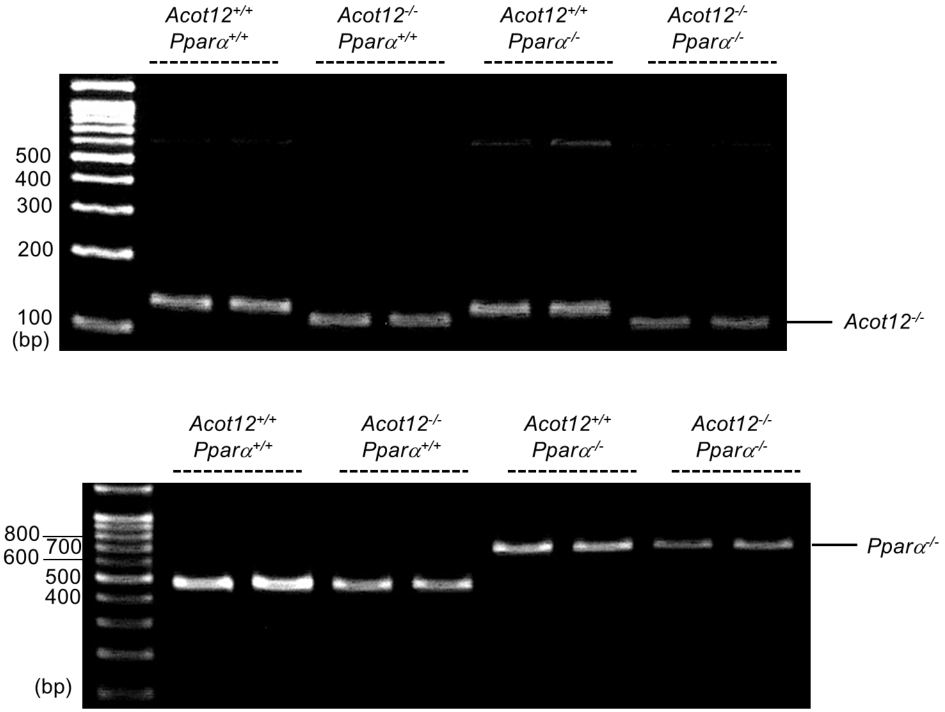

Supplementary Fig. 2. Genotyping of *Acot12*$^{+/+}$*Ppar*$\alpha^{+/+}$, *Acot12*$^{-/-}$*Ppar*$\alpha^{+/+}$, *Acot12*$^{+/+}$*Ppar*$\alpha^{-/-}$, and *Acot12*$^{-/-}$*Ppar*$\alpha^{-/-}$ kidneys.

Supplementary Fig. 3

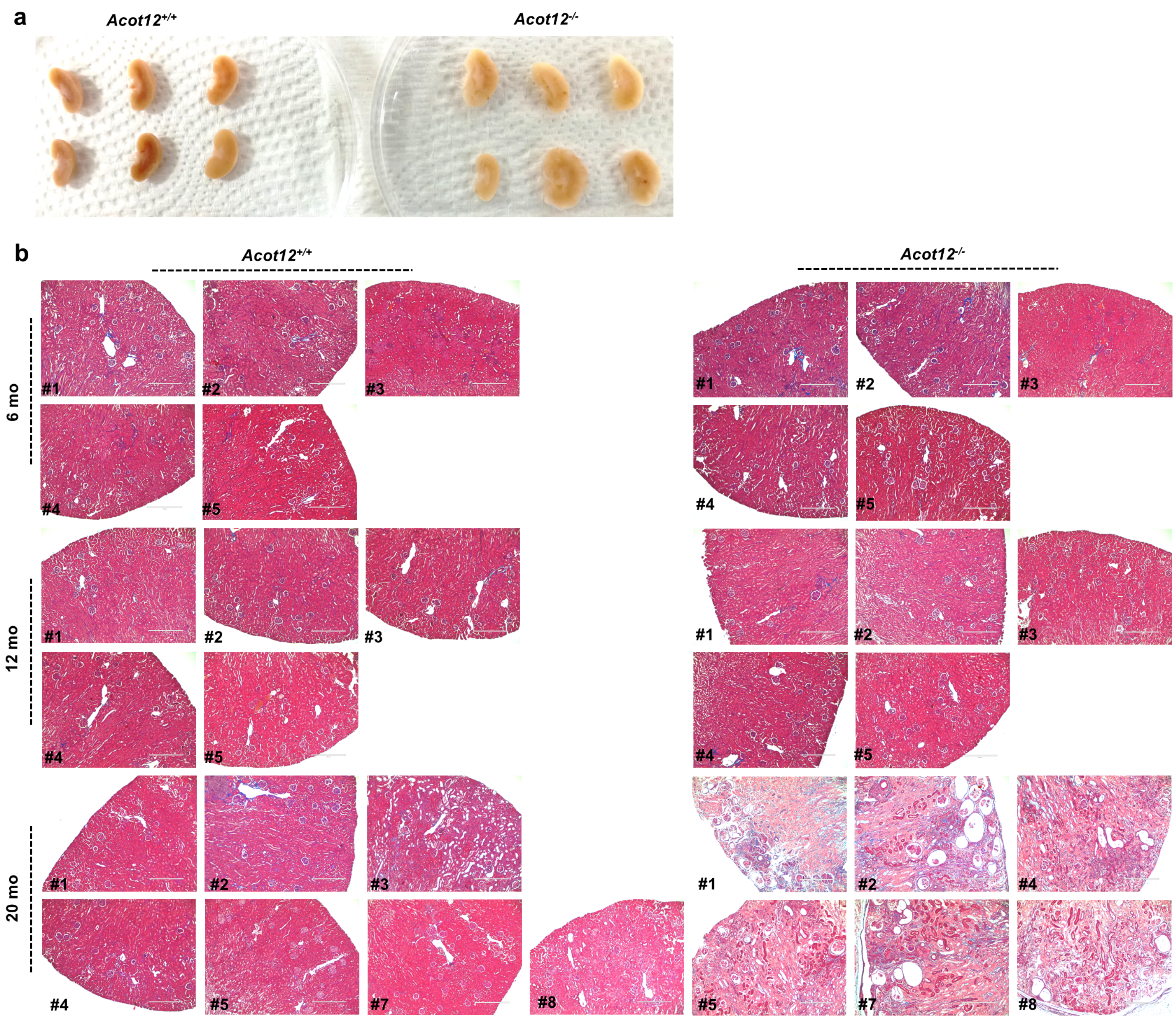

Supplementary Fig. 3. Aging kidneys of *Acot12*$^{+/+}$ and *Acot12*$^{-/-}$ mice. a 6 kidneys of 20-month-old *Acot12*$^{+/+}$ or *Acot12*$^{-/-}$ mice. **b** Masson's Trichrome staining of 6-, 12-, and 20-month-old *Acot12*$^{+/+}$ and *Acot12*$^{-/-}$ kidney used in this study. Scale bar = 400 μm.

Supplementary Fig. 4

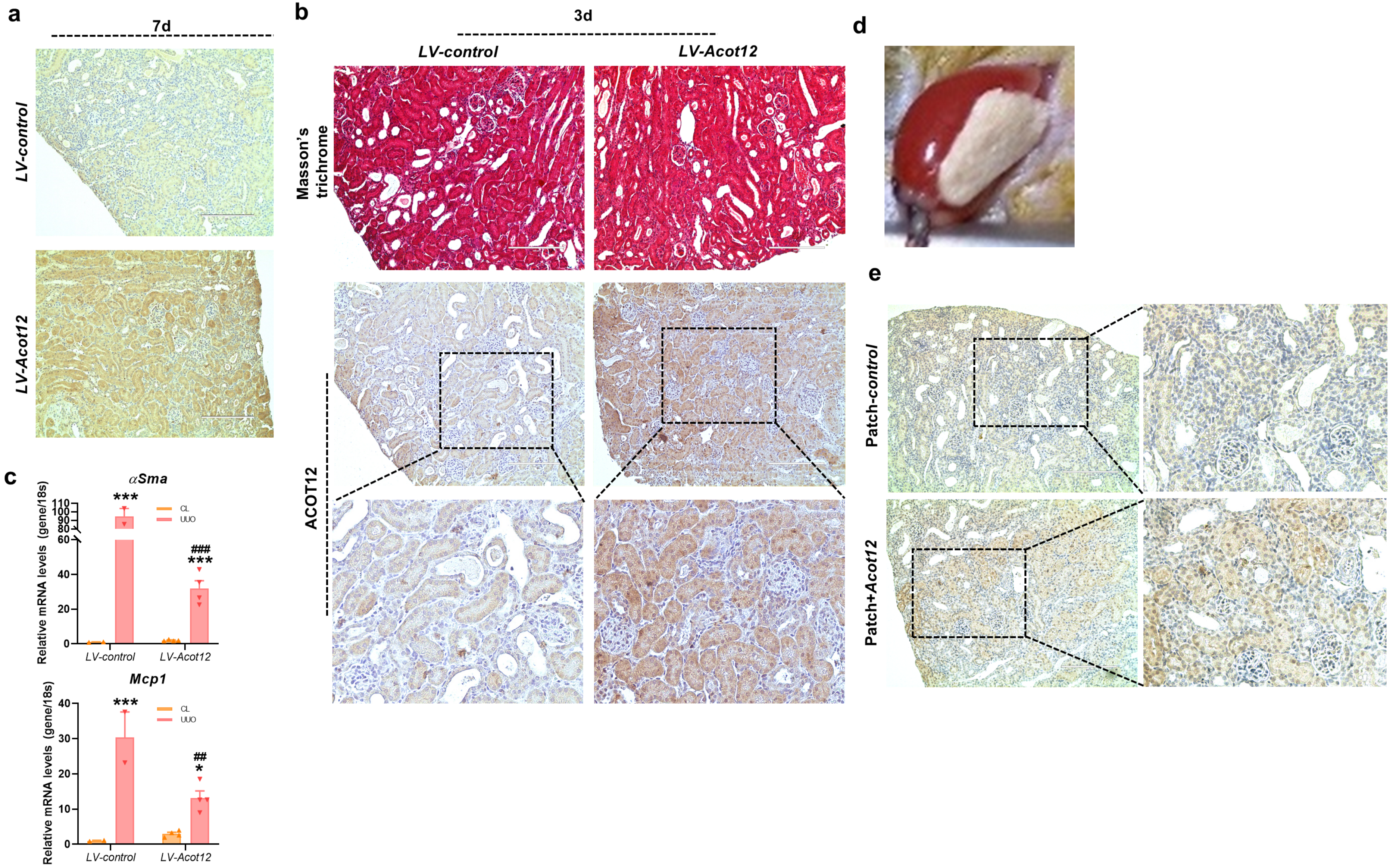

Supplementary Fig. 4. Lentivirus-mediated and chitosan patch-mediated ACOT12 overexpression. a Immunohistochemical staining of ACOT12 with parenchymal injection of lentivirus containing *Acot12* construct (*LV-Acot12*) into *Acot12*$^{-/-}$ kidneys. Control lentivirus (*LV-control*) was used as negative control an mice were sacrificed on day 7. Scale bar = 200 μm. **b** Masson's trichrome and immunohistochemical staining of PLIN2 and ACOT12 in *Acot12*$^{-/-}$ UUO kidneys with parenchymal injection of *LV-Acot12*. Control lentivirus was used as a negative control and mice were sacrificed on day 3. Scale bar = 200 μm. **c** The expression level of fibrotic and inflammatory genes with parenchymal injection of *LV-Acot12*. Control lentivirus was used as a negative control and mice were sacrificed on day 3 (n= 3-4 per group). **d** Picture of kidney attached with chitosan patch conjugated with *Acot12* expression vector (Patch-*Acot12*) during UUO operation. A control expression vector was conjugated into chitosan patch (Patch) and used as negative control. **e** Immunohistochemical staining of ACOT12 with Patch- or Patch-*Acot12*-attached *Acot12*$^{-/-}$ kidneys. Scale bar = 200 μm. * $P < 0.05$; ** $P < 0.01$; *** $P < 0.001$ per control CL. # $P < 0.05$; ## $P < 0.01$; ### $P < 0.001$ per control UUO.

Supplementary Fig. 5

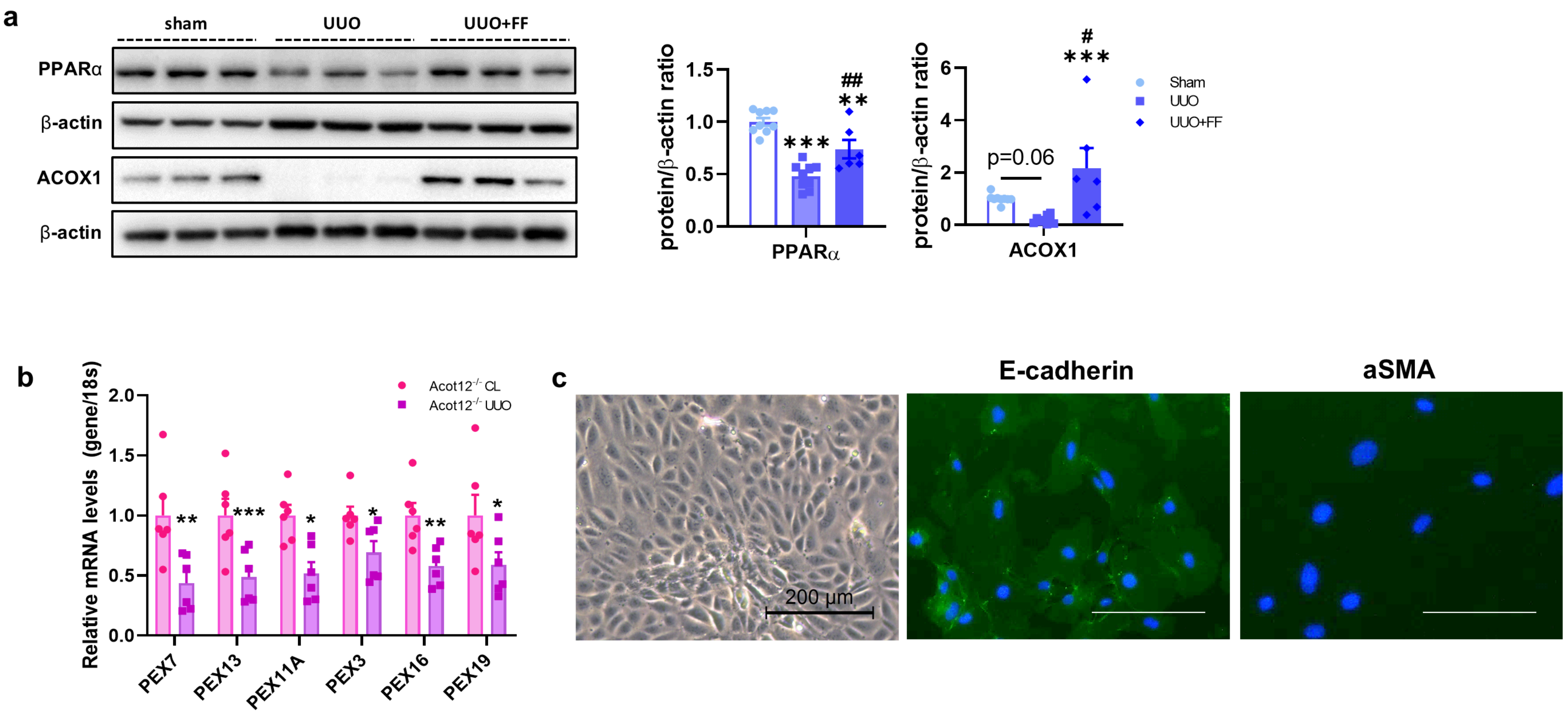

Supplementary Fig. 5. a Western blot of PPARα and its downstream target ACOX1 in *Acot12*$^{-/-}$ UUO kidney with fenofibrate treatment (n=6-9 per group). **b** The expression level of peroxisome biogenesis genes in *Acot12*$^{-/-}$ UUO kidney (n=6). **c** Immunofluorescence characterization of PTECs, showing E-cadherin as an epithelial marker and αSMA as a mesenchymal marker. Scale bar = 200 μm. * *P* < 0.05; ** *P* < 0.01; *** *P* < 0.001 per sham or CL. # *P* < 0.05; ## *P* < 0.01 per UUO.

Supplementary Fig. 6

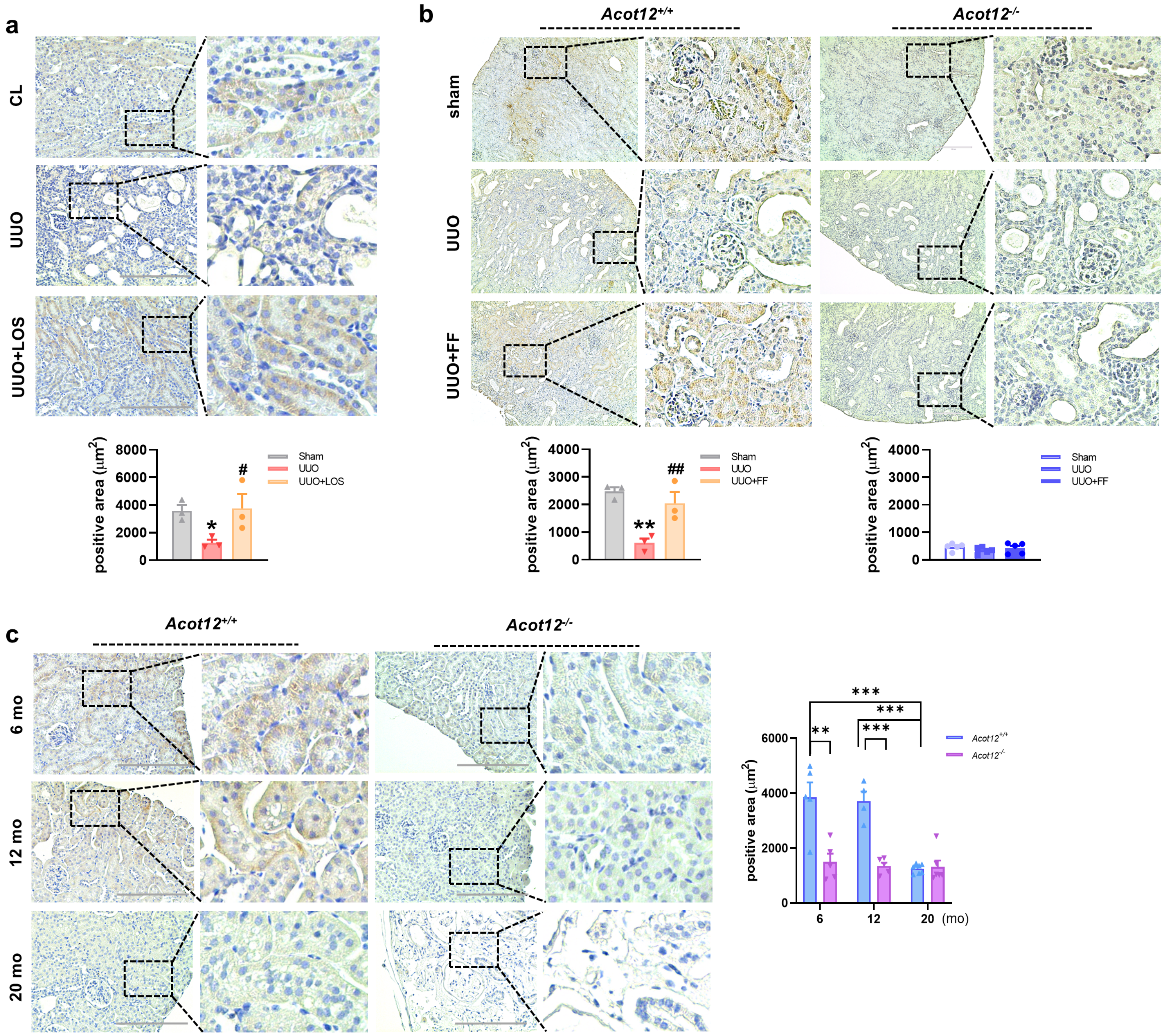

Supplementary Fig. 6. The expression level of ACBD5 expression in various kidneys used in this study. a Immunohistochemical staining of ACBD5 in UUO kidney with or without treatment of losartan. Scale bar = 200 μm. **b** Immunohistochemical staining of ACBD5 in *Acot12*$^{+/+}$ or *Acot12*$^{-/-}$ UUO kidneys with or without oral administration of fenofibrate. Scale bar = 200 μm. **c** Immunohistochemical staining of ACBD5 in 6-, 12-, and 20-month-old *Acot12*$^{+/+}$ kidneys. Scale bar = 200 μm. * $P < 0.05$; ** $P < 0.01$; *** $P < 0.001$ per sham or indicated group. # $P < 0.05$; ## $P < 0.01$ per UUO.

Supplementary Fig. 7

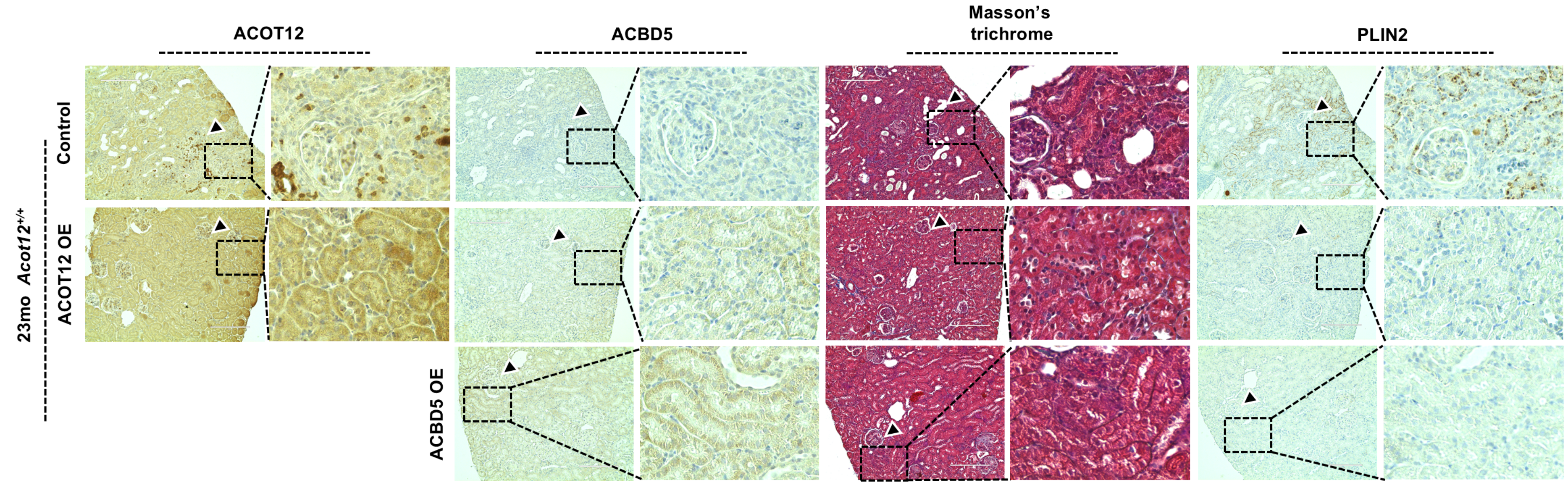

Supplementary Fig. 7. The immunostaining images of ACOT12, ACBD5, PLIN2 and Masson's trichrome in Acot12- or Acbd5-overexpressed 23-month-old *Acot12*$^{+/+}$ kidneys. Scale bar = 200 μm.

SUPPLEMENTARY TABLE

Supplementary Table1. qRT-PCR primer sequences

Gene	Forward primer	Reverse primer
Abcd1	GTACAGTCTGGTGTCCATGTAG	GGGTGTGATGATAGGGATGTT
Acaca	AGCCAGAAGGGACAGTAGAA	CTCAGCCAAGCGGATGTAAA
Acbd5	AAAAGATCGCTCTTGTGCTCATTAG	AAAGCTAATGAGCACAAGAGCGATC
Acly	CGGGAGGAAGCTGATGAATATG	GTCAAGGTAGTGCCCAATGAA
Acot12	CCACCACCTTGGAGAAGATAAA	GTGAGGTCAGACAAGAGATGATAAG
Acss1	GAGCATGAGCAGTGAAGACA	CATGGCGGCATACAGTAGATAG
Acss2	CACCTTCTGGCAAACAGAAAC	CTACACCGAAGAATGGGAAAGA
aSMA	GTCCCAGACATCAGGGAGTAA	TCGGATACTTCAGCGTCAGGA
Col1a1	GAACATCACCTACCACTGCA	GTTGGGATGGAGGGAGTTTA
Fasn	AAGTTGCCCGAGTCAGAGAA	TTCCAGACCGCTTGGGTAAT
Mcp1	CTTCTGGGCCTGCTGTTCA	CCAGCCTACTCATTGGGATCA
Pex1	GGAGATGGAGAATGTGGCTTAG	CCAAAGTAGAGCCGGTACATATT
Pex10	ATGGCTGGAATGGAGGAAAG	CTTGGATGATCCCAACGTACTC
Pex11a	TCCTTCCTCCTCCTCCTATTC	GCCGAGATTGGACTTGTAGAT
Pex12	CAGAAGCGTTGGTGAGAAGATA	CAGGAAGAATACACCCACAGAG
Pex13	GTTATCCTTGGCGGTCCTTATC	TCTTGCAACTACGTGGTCATC
Pex16	ACTGTCTGGTGTGGTAGATATG	GTGAGCGTAGCAGGTAGTATAG
Pex19	GCTAGATCTCATGCAGCAGTTA	TTGAGAGCATCCAGGTCAAAG
Pex2	CCCACAATGCCTCACACTAT	CACTTCGGGCACGTAAAGTA
Pex26	CTGGAGCTGTGCATCCTTT	AGGAAGGCCCTGGTTATCT
Pex3	CCTACAGCATGGTAACTCCATAA	GTGTCTCACTGCAAACTGAATG
Pex6	CTGACTGGTGCAGATCTCTATTC	CAGCTCTAGCCCTTCCTCTA
Pex7	CATGTCCTTGTCACCTGTAGTG	CTCCTGCGTGTGCTCTTTAT
Rn18s	CCAGTAAGTGCGGGTCATAAG	GGCCTCACTAAACCATCCAA
Scd1	CAACTTCACCACGTTCTTCATC	CCCGTCTCCAGTTCTCTTAATC
Tgfb	CTTTAGGAAGGACCTGGGTT	CAGGAGCGCACAATCATGTT
